# Effect of 4 years of growth hormone therapy in children with Noonan syndrome in the American Norditropin Studies: Web-Enabled Research (ANSWER) Program® registry

**DOI:** 10.1186/1687-9856-2012-15

**Published:** 2012-06-08

**Authors:** Peter A Lee, Judith Ross, John A Germak, Robert Gut

**Affiliations:** 1 Department of Pediatrics, The Milton S. Hershey Medical Center, Penn State College of Medicine, Hershey, PA, USA; 2 DuPont Hospital for Children, Department of Pediatrics, Thomas Jefferson University, Philadelphia, PA, USA; 3 Novo Nordisk Inc., Department of Clinical Development, Medical and Regulatory Affairs, Princeton, NJ, USA

**Keywords:** Growth hormone, Noonan Syndrome, Somatropin, Short stature, Body height, Treatment outcome

## Abstract

**Background:**

Noonan syndrome (NS) is a genetic disorder characterized by phenotypic features, including facial dysmorphology, cardiovascular anomalies, and short stature. Growth hormone (GH) has been approved by the United States Food and Drug Administration for short stature in children with NS. The objective of this analysis was to assess the height standard deviation score (HSDS) and change in HSDS (ΔHSDS) for up to 4 years (Y4) of GH therapy in children with NS.

**Methods:**

The American Norditropin Studies: Web-Enabled Research (ANSWER) Program®, a US-based registry, collects long-term efficacy and safety information on patients treated with Norditropin® (somatropin rDNA origin, Novo Nordisk A/S) at the discretion of participating physicians. A total of 120 children (90 boys, 30 girls) with NS, naïve to previous GH treatment, were included in this analysis.

**Results:**

The mean (SD) baseline age of subjects (n = 120) was 9.2 (3.8) years. Mean (SD) HSDS increased from –2.65 (0.73) at baseline to –1.32 (1.11) at Y4 (n = 17). Subjects showed continued increase in HSDS from baseline to Y4 without significant differences between genders at Y1 or Y2. The mean (SD) GH dose was 47 (11) mcg/kg/day at baseline and 59 (16) mcg/kg/day at Y4. There was a negative correlation between baseline age and ΔHSDS at Y1 (R = –0.3156; P = 0.0055) and Y2 (R = –0.3394; P = 0.017). ΔHSDS at Y1 was significantly correlated with ΔHSDS at Y2 (n = 37; R = 0.8527, P < 0.0001) and Y3 (n = 20; R = 0.5145; P = 0.0203), but not Y4 (n = 12; R = 0.4066, P = 0.1896).

**Conclusions:**

GH treatment-naïve patients with NS showed continued increases in HSDS during 4 years of treatment with GH with no significant differences between genders up to 2 years. Baseline age was negatively correlated with ΔHSDS at Y1 and Y2. Whether long-term therapy in NS results in continued increase in HSDS to adult height remains to be investigated.

**Trial registration:**

ClinicalTrials.gov NCT01009905

## Introduction

Noonan syndrome (NS), a genetic disorder first described by Noonan and Ehmke in 1963 [1], is characterized by phenotypic features including facial dysmorphology, cardiovascular anomalies, and short stature [2]. Patients with NS are typically born with appropriate size for gestational age, but reach median adult heights of only 162.5 cm and 152.7 cm for men and women, respectively, values that are approximately 2 SDS below the normal population [3]. While the etiology of short stature in NS patients is not definitively known, growth hormone (GH) therapy has been shown to improve growth rates [4]. This improvement in both growth rates and adult height is attributed, at least in part, to increased production of insulin-like growth factor 1 (IGF-1) [5]. Treatment with GH therapy has been shown to normalize height standard deviation scores (HSDSs) during childhood for patients with NS [6,7].

Despite the successful gain in height associated with GH therapy, response to treatment often varies. For many patients, factors such as GH dose and age at start of treatment may affect the outcome of GH therapy [8]. Furthermore, the success of GH therapy for patients with NS may also be influenced by the genetic causes of the disorder, although genetic mutations have not been identified in all patients with NS [9]. Currently identified genetic mutations explain approximately 60% of NS cases [10]. Genetic mutations associated with the NS phenotype are involved in the Ras/MAPK (mitogen-activated protein kinase) signal transduction pathway [10,11]. Several candidate genes involved in the Ras/MAPK signaling pathway have been identified, with the protein tyrosine phosphatase non-receptor type 11 gene (*PTPN11*) responsible for the greatest number of cases [10,11].

In 2007, the United States (US) Food and Drug Administration (FDA) approved the use of GH for short stature in children with NS [12]. While this FDA approval is relatively recent, NS patients have been receiving GH therapy in clinical trials for a number of years, and the results of these trials have been published [2,4,7,13-18]. Since 2002, the American Norditropin Studies: Web-Enabled Research (ANSWER) Program® registry, which utilizes the enhanced research Web-based platform, NovoNet®, has been collecting long-term efficacy and safety data on patients treated with Norditropin® (somatropin rDNA origin, Novo Nordisk A/S) in the United States. The use of Norditropin in patients within the ANSWER Program and participation in the registry is at the discretion of the contributing physician investigators and patient informed consent and includes additional diagnostic conditions that warrant treatment with GH. Also, completeness of data concerning patients (e.g. background histories and physical examination data, and additional findings from history and examination in addition to short stature, such as cardiac and skeletal problems) may vary. Recently, data from the ANSWER Program registry was used to assess the impact of gender, puberty, and age on change in height standard deviation scores (ΔHSDSs) following 2 years of GH treatment across many diagnostic categories, including GH deficiency, multiple pituitary hormone deficiency, Turner syndrome, small for gestational age, NS, and idiopathic short stature [19]. The study showed that increase in HSDS could be achieved for all diagnostic categories, particularly when treatment was initiated at an early age. In the present analysis, data from the ANSWER Program registry are assessed to determine HSDS and ΔHSDS during up to 4 years of GH therapy in children with NS.

## Objective and methods

The ANSWER Program is a US-based registry that has collected long-term efficacy and safety information for GH treatment-naïve and non-naïve patients treated with Norditropin since 2002 (NCT01009905). Patients’ medical histories and physical examination data were entered by participating physician investigators using the NovoNet Web-based data entry tool. At the initial visit, study investigators collected baseline HSDS, weight, pretreatment bone age, maximal stimulated serum GH concentration, and serum IGF-1 concentrations. The data collected at follow-up clinical visits included GH dose/frequency, height, weight, IGF-1 concentration, and bone age. Dosing was determined by the treating physician.

Although both GH-naïve and non-naïve patients are included in the ANSWER Program registry, data from only GH-treatment-naïve patients (aged ≤ 18 years) with NS were included in the current analysis. Potential subjects were excluded from the analysis if key variables had baseline or subsequent values that were deemed physically or chronologically implausible. Cross-sectional data from baseline, at year 1 (Y1), year 2 (Y2), year 3 (Y3), and year 4 (Y4) were analyzed. Data at each post-baseline time point were collected within a ±3-month window. Baseline data were summarized, including gender, age, HSDS, IGF-1 standard deviation score (IGF-1 SDS), bone age, and maximal stimulated serum GH concentration. HSDS (z score) was calculated according to the standard formulas provided by the Centers for Disease Control and Prevention [20]. Target height was calculated using the following formula: [average of the parents’ heights] + 6.5 cm for males and [average of the parents’ heights] − 6.5 cm for females. Target height SDS is calculated according to the standard formulas provided by the CDC. HSDS corrected for target height, [height SDS at each visit] − [target height SDS], was determined annually. Change in HSDS (ΔHSDS) was compared between gender groups using least squares means estimates from an analysis of covariance (ANCOVA) model with gender as fixed effect and baseline HSDS value as a covariate. For analyses of age at start of treatment, age ranges were defined to be <11 or ≥11 years of age for boys, and <10 or ≥10 years of age for girls. These ages were chosen so that both younger age categories would be comprised primarily of pre-pubertal individuals. Statistical comparisons of ΔHSDS between age groups stratified by gender were conducted using *t*-tests. Linear regression was performed to identify the relationship between ΔHSDS after 1 or 2 years of GH treatment and age at start of treatment. Data following 3 or 4 years of treatment were not analyzed for logistic regression due to a limited number of patients. The corresponding Pearson correlation coefficients (R) were calculated along with the p values for testing their significance. Mean GH dose at each time point was summarized. Analyses were performed to evaluate (1) whether ΔHSDS during the first year of therapy was predictive of ΔHSDS at later time points (linear regression analysis), (2) whether baseline IGF-I or baseline IGF-I SDS was predictive of ΔHSDS (linear regression analysis), and (3) whether ΔHSDS differed for GH-deficient versus GH-sufficient patients (deficient GH status was defined as GH peak at baseline <10; ΔHSDS was compared between groups using *t*-tests).

## Results

### Baseline demographics and patient disposition

The ANSWER Program registry contained information for 120 children (90 boys and 30 girls) with NS. The mean (SD) baseline age of all children with NS was 9.2 (3.8) years; the mean age for boys and girls was similar. All patients in the younger age groups (ie, boys <11 years and girls <10 years) were Tanner stage 1. Other characteristics are summarized in Table 1. Mean (SD) maximal GH level was 11.7 (8.4) ng/ml (n = 34) with range of 0.8 to 39.3 ng/ml, while mean (SD) IGF-1 SDS of –3.0 (1.5) indicated low IGF-1 levels (n = 73). The numbers of patients that completed 1, 2, 3, and 4 years of treatment were 76, 49, 31, and 17, respectively.

**Table 1:** Baseline Demographics

**Characteristic**	**N**	**Mean (SD)**	**Median**	**Range**
Age, years				
All subjects	120	9.2 (3.8)	9.6	1.6, 16.9
Boys	90	9.20 (4.09)	9.6	1.6, 16.9
Girls	30	9.24 (2.98)	9.6	2.8, 14.2
HSDS				
All subjects	120	–2.6 (0.7)	–2.6	–4.5, –1.2
Boys	90	–2.6 (0.74)	–2.44	–4.5, –1.2
Girls	30	–2.9 (0.67)	–2.88	–4.1, –1.7
Target HSDS	99	–0.3 (0.9)	–0.2	–3.8, 2.0
IGF-1 SDS	73	–3.0 (1.5)	–2.8	–8.2, 0.6
Bone age, years	93	7.7 (3.8)	7.8	0.5, 15.5
Maximal GH, ng/ml	34	11.7 (8.4)	10.0	0.8, 39.3
BMI	120	16.4 (2.0)	16.1	13.2, 26.9
Weight, kg	120	23.9 (9.4)	22.8	8.3, 51.6

*BMI*, body mass index; *GH*, growth hormone; *HSDS*, height standard deviation score; *SD*, standard deviation.

### The effects of GH treatment on HSDS

**Figure 1:**
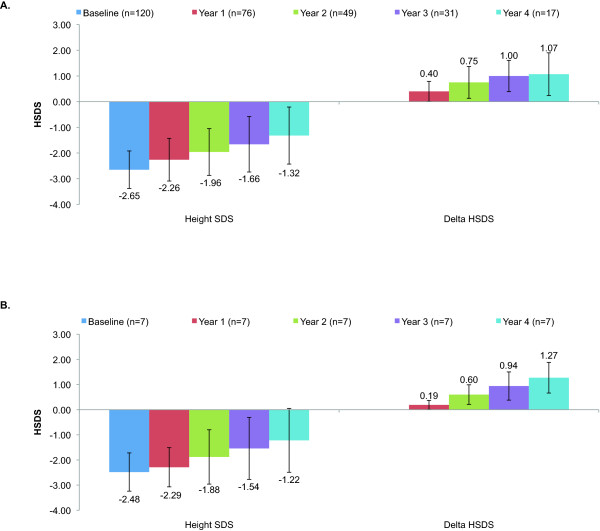
**Mean HSDS Change at Year 1, Year 2, Year 3, and Year 4: (A) Cross-sectional Data and (B) Longitudinal Data.** HSDS, height standard deviation score.

The mean (SD) GH dose for the NS patients was 47 (11) mcg/kg/day at baseline, 52 (12) mcg/kg/day after 1 year of treatment, 49 (15) mcg/kg/day after 2 years of treatment, 54 (19) mcg/kg/day after 3 years of treatment, and 59 (16) mcg/kg/day after 4 years of treatment. Among patients with 4 years of longitudinal data (n = 7), the mean (SD) GH dose was 46 (3) mcg/kg/day at baseline, 53 (11) mcg/kg/day at 2 years, 57 (15) at 2 years, 58 (19) mcg/kg/day at 3 years, and 63 (16) at 4 years. The overall effects of GH treatment on patients with NS are summarized in Figure 1. Cross-sectional data show that mean (SD) HSDS increased from –2.65 (0.73) at baseline to –1.32 (1.11) after 4 years of treatment (Figure 1A). Mean (SD) HSDS corrected for target height, was –2.4 (1.02) at baseline, –2.0 (1.09) at Y1 (n = 64), –1.5 (1.22) at Y2 (n = 42), –1.5 (1.24) at Y3 (n = 29), and –1.0 (1.50) at Y4 (n = 14). Among the 7 patients for whom 4 years of longitudinal data were available, mean (SD) HSDS was –2.48 (0.76) at baseline, –2.29 (0.78) after 1 year of treatment, –1.88 (1.08) after 2 years of treatment,–1.54 (1.23) after 3 years of treatment, and –1.22 (1.24) after 4 years of treatment (Figure 1B). Following 4 years of GH treatment 12 of 17 patients (71%) achieved height normal for age and gender (defined as HSDS > –2SD).

**Figure 2:**
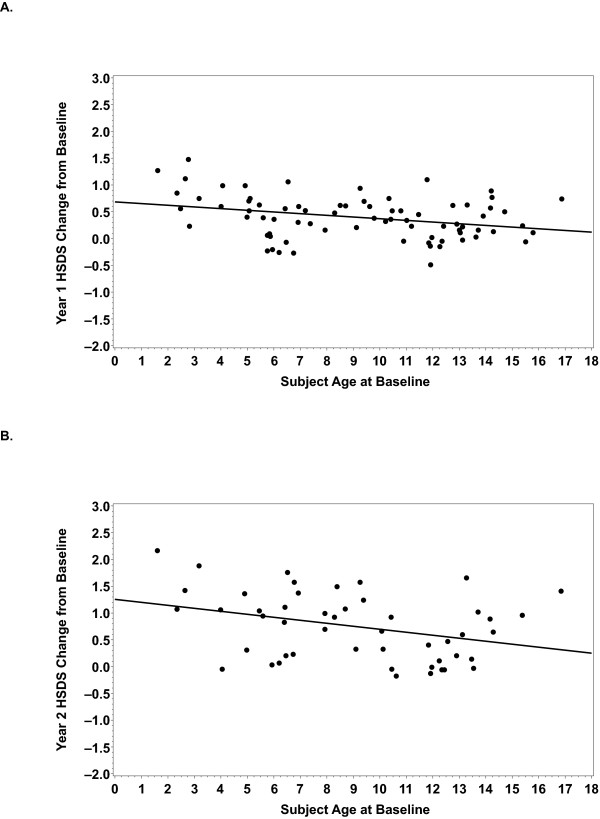
**Linear Regression of ΔHSDS at Year 1 and Year 2 on Baseline Age**. At both (**A**) Year 1 (n = 76; correlation = -.3156; P = 0.0055) and (**B**) Year 2 (n = 49; correlation = -.3394; P = 0.017), there was a significant negative correlation between ΔHSDS and baseline age (ie, ΔHSDS decreased as baseline age increased).

Patient gender did not significantly affect the outcome of GH therapy for patients with NS. Mean (SD) HSDS increased progressively from baseline for both boys and girls. Comparisons from the ANCOVA model showed no significant differences between boys and girls after 1 or 2 years treatment. On the contrary, patient age at onset of therapy did affect outcome of GH therapy. There was a significant negative correlation between baseline age and change in HSDS after 1 year (n = 76; correlation coefficient R = –0.3156; P = 0.0055) and 2 years of treatment (n = 49; R = –0.3394; P = 0.017) (Figure 2). Among boys, there was a significantly greater ΔHSDS from baseline for the younger age group versus the older age group after 1 and 2 years of treatment; no significant differences were observed among girls at either time point, which may reflect the lower number of female patients (Table 2).

**Table 2:** HSDS Change from Baseline Stratified by Age at Treatment Start and by Gender

	**Boys**					**Girls**				
	**<11 years**		**≥11 years**			**<10 years**		**≥10 years**		
	**N**	**Mean (SD)**	**N**	**Mean (SD)**	**P value**	**N**	**Mean (SD)**	**N**	**Mean (SD)**	**P value**
Y1	32	0.53 (0.36)	22	0.24 (0.34)	0.0046	12	0.42 (0.51)	10	0.29 (0.38)	0.5159
Y2	25	0.94 (0.65)	13	0.47 (0.50)	0.0284	6	0.77 (0.55)	5	0.49 (0.67)	0.4726
Y3	16	0.92 (0.65)	10	1.01 (0.65)	0.7168	2	1.24 (0.33)	3	1.24 (0.45)	0.9968
Y4	6	1.15 (0.93)	8	0.90 (0.88)	0.6082	2	1.34 (0.78)	1	1.52 (—)	—

P values are for comparisons between age groups for each year.

*HSDS*, height standard deviation score; *SD*, standard deviation; Y1, year 1; Y2, year 2; Y3, year 3; Y4, year 4.

**Figure 3:**
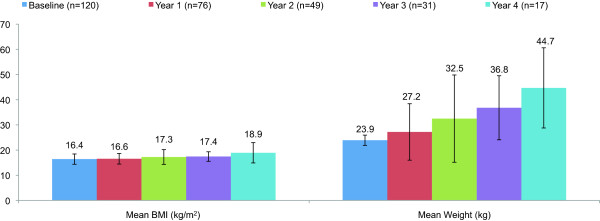
**Mean BMI and Weight Change at Year 1, Year 2, Year 3, and Year 4 (Cross-sectional Data).** BMI, body mass index/

Although the number of children with post-baseline IGF-1 SDS data was limited, mean (SD) IGF-1 SDS increased from a value of –2.96 (1.55) at baseline (n = 73) to –1.35 (2.31) after 1 year of treatment (n = 17), –0.87 (2.79) after 2 years of treatment (n = 12),–0.02 (1.08) after 3 years of treatment (n = 7), and 0.22 (2.62) after 4 years of treatment (n = 5). Mean (SD) weight increased from baseline following 4 years of treatment, whereas body mass index (BMI) remained stable (Figure 3). The small magnitude of the change in BMI suggests that increases in weight were proportional to increases in height.

Several analyses were performed to examine early potential predictors of later response. ΔHSDS at Year 1 was significantly correlated with ΔHSDS at Year 2 (n = 37; R = 0.8527, P < 0.0001) and Year 3 (n = 20; R = 0.5145; P = 0.0203), but not Year 4 (n = 12; R = 0.4066, P = 0.1896). Neither baseline IGF-1 levels nor baseline IGF-1 SDS were predictive of ΔHSDS (data not shown). GH-deficient patients (n = 16) tended to have greater ΔHSDS than GH-sufficient patients (n = 18); the difference was statistically significant only at Year 2 (GH-deficient: n = 7, mean [SD] ΔHSDS: 1.08 [0.66] vs GH-sufficient: n = 13, mean [(SD] ΔHSDS: 0.40 [0.56]; P = 0.0266).

## Discussion

Results from this 4-year analysis of GH therapy of NS subjects from the ANSWER Program registry demonstrate an increase in HSDS over the course of treatment. The mean HSDS (n = 120) at start of treatment was –2.6. By the end of 3 years of GH therapy, mean HSDS had increased to –1.66 (n = 31), and by the end of 4 years to –1.32 (n = 17). This increase in mean HSDS is similar to data from a clinical trial conducted by MacFarlane et al., in which HSDS (SD) of NS patients (n = 23) increased from –2.7 (0.4) at the start of GH therapy to –1.9 (0.9) following 3 years of treatment [15]. Our findings are consistent with, and perhaps better than, results from the National Cooperative Growth Study (NCGS), which reported an increase in HSDS from –3.3 (0.9) at baseline to –2.4 (1.1) at 3 years and –2.1 (1.2) at 4 years (n = 42) [4], and to the Pharmacia & Upjohn International Growth Study (KIGS), which reported an increase from –2.9 (0.7) at baseline to approximately –2.3 at 4 years (n = 25) [14]. We also found an increase in HSDS corrected for target height from –2.4 (1.02) at baseline to –1.0 (1.50) at Y4.

Furthermore, for patients for whom longitudinal data were available (n = 7), mean HSDS increased consistently over the 4 years of treatment, indicating that GH therapy of NS patients results in sustained growth over multiple years of treatment. The yearly incremental change in mean HSDS for these patients was 0.19, 0.41, 0.34, and 0.32 for Y1, Y2, Y3, and Y4 respectively. This trend of sustained growth is consistent with a previous registry-based study conducted by Romano et al. In 1996, the group analyzed data of 150 children with NS who were enrolled in the NCGS [4]. For the 42 children in the study who were monitored for at least 4 years of GH therapy, yearly incremental change in mean HSDS was 0.5, 0.2, 0.2, and 0.3 for Y1, Y2, Y3, and Y4 of treatment, respectively, with some patients exceeding their predicted height by the end of treatment..

However, even after 4 years of GH therapy, 29% (5/17) of patients remained short for age and gender (as defined by HSDS < –2 SD). Possible reasons for this could include an innate resistance to GH therapy among some with NS or advanced bone age/chronologic age at treatment start. Without treatment, height in NS follows the 3rd percentile during the first several years of life, and then generally declines further at puberty, with mean final height approximately 2 SDS below normal limits [3,21].

The mean increase in HSDS was similar for boys and girls in this study, suggesting that gender does not significantly affect the outcome of treatment. This trend differs from previous clinical trial studies in which gender was shown to affect the outcome of GH therapy in patients with NS [6,16].

In contrast to what was observed in this analysis, previous registry-based studies have assessed the short- and long-term effects of GH therapy in patients with NS and found that the increase in height of NS patients treated with GH therapy is highest after 1 year, but wanes in subsequent years of treatment. In 2001, Kirk et al. conducted an analysis of NS patients involved in the KIGS study [14]. The yearly incremental change in HSDS for these patients waned significantly after 1 year of treatment. Mean HSDS increased by 0.3 over the first year, but further increase was not observed over the next 4 years. A similar trend was observed by Raaijmakers, et al., who analyzed the growth response in 402 NS patients enrolled in the KIGS database who were treated with GH therapy [18]. After 1 year of treatment, mean HSDS increased by 0.54, but the incremental increases were significantly lower (0.13 and 0.13) following years 2 and 3 of GH therapy. Finally, in the clinical trial conducted by MacFarlane et al., ΔHSDS was 0.5 during the first year of treatment, but the yearly incremental increase in mean HSDS dropped to 0.1 and 0.2 for the second and third years of treatment, respectively. It was confirmed in the MacFarlane clinical trial that the lower incremental increase in HSDS for years 2 and 3 of treatment could not be attributed to reduced growth rate caused by non-adherence to therapy [15]. Potential explanations for waning growth may be related to older age at treatment onset, GH sufficiency status, GH dosage, and the presence of other genetic findings specific to NS. The presence of a specific mutation may be a factor in the response to GH treatment, although the type of mutation does not necessarily correlate with the severity of short stature or the patient’s response to GH therapy [16,22-25]. In the current study, the incremental gain in HSDS among the 7 patients for whom longitudinal data were available was lowest during Year 1. The mean GH dose among these patients increased each year, which may explain, in part, why the incremental gains were higher after Year 1.

The mean (SD) weight determined for the patients enrolled in the ANSWER Program registry increased from 23.9 (9.4) kg at baseline to 44.7 (15.9) kg following 4 years of treatment, whereas body mass index (BMI) remained stable. The small magnitude of the change in BMI suggests that increases in weight were proportional to increases in height, indicating that GH therapy for NS patients did not significantly impact body composition in ways unrelated to linear growth. Although the number of children with post-baseline IGF-1 SDS data was limited, analysis of the available data showed that mean (SD) IGF-1 SDS increased. Previous studies have indicated a positive linear relationship between the change in IGF-1 and ΔHSDS for patients undergoing GH treatment [26].

In addition to sustained growth over the course of GH treatment, and a positive correlation between ΔHSDS at Year 1 and ΔHSDS at Years 2 and 3, analysis of data from boys and girls in the ANSWER Program registry also showed that a negative correlation exists between the age at the start of treatment and ΔHSDS (Figure 2). That is, ΔHSDS decreased as age at initiation of treatment increased. In an analysis using age 11 as a cutoff for younger versus older age, boys who began treatment before the age of 11 years showed a mean ΔHSDS of 0.53 after 1 year of treatment and 0.94 after 2 years (Table 2). On the other hand, when treatment was initiated for boys at an age greater than 11 years, mean ΔHSDS was only 0.24 after 1 year (P = 0.046) and 0.47 after 2 years (P = 0.0284). The same trend was observed for girls in this study, although differences were not statistically significant. Although the age cut-offs of 11 years for boys and 10 years for girls did correspond to baseline pubertal stage (ie, all patients in the younger age groups were pre-pubertal), the relative contribution of the accelerated growth rate during the pubertal growth spurt is unclear. A similar pattern was observed by Romano et al. in data from the NCGS [7], which showed that greater near adult height (NAH) for NS patients was associated with earlier initiation and longer duration of GH therapy.

These results emphasize the importance of early diagnosis and initiation of therapy for optimal height outcomes. However, diagnosing NS is a difficult task, even for many specialists, due to the fact that it is primarily based on clinical features. Therefore, the Noonan Syndrome Support Group recently coordinated a conference comprised of professionals with extensive knowledge of various aspects of the disease to develop guidelines for the diagnosis and management of the disorder [10]. These guidelines provide pediatricians and other healthcare professionals with a comprehensive description of genetic factors associated with NS and key clinical features of the disorder.

One limitation of the current study is the lack of data regarding the underlying genetic defect, particularly the presence or absence of the *PTPN11* mutation, responsible for causing NS in these patients. Nonetheless, this analysis provides valuable information, such as expectations for treatment outcomes and the potential to optimize growth by initiating GH treatment early, which can help to guide clinicians who treat patients with NS.

In conclusion, this analysis of the ANSWER Program registry shows that continued increase in HSDS after 4 years of treatment with GH could be achieved in GH-naïve subjects with NS, with no significant differences in treatment outcome between genders at years 1 and 2. The data also show that baseline age was negatively corty related with ΔHSDS following 1 and 2 years of treatment. Whether longer-term therapy will have a beneficial effect on adult height remains to be investigated.

## Competing interests

PL: Has participated in patient registries of growth hormone treated patients supported by Genentech, Eli Lilly and Pfizer.

JR: Has participated in patient registries of growth hormone treated patients supported by Genentech, Eli Lilly and Pfizer. JR is a consultant for Eli Lilly, Pfizer and Novo Nordisk Inc.

JG: Is an employee of Novo Nordisk Inc and as such has received stock/stock options.

RG: Is an employee of Novo Nordisk Inc and as such has received stock/stock options.

## Authors’ contributions

PL and JR were primary investigators and interpreted the data, critically reviewed and suggested revisions for manuscript drafts, and approved final draft. JG and RG contributed to the acquisition of data, provided analysis and interpretation the data, critically reviewed draft, and approved final draft. All authors read and approved the final manuscript.
